# The complete chloroplast genome of the national tree of Peru, quina (*Cinchona officinalis* L., Rubiaceae)

**DOI:** 10.1080/23802359.2021.1969697

**Published:** 2021-09-06

**Authors:** Carlos I. Arbizu, Rubén D. Ferro-Mauricio, Julio C. Chávez-Galarza, Juan Carlos Guerrero-Abad, Héctor V. Vásquez, Jorge L. Maicelo

**Affiliations:** a Dirección de Desarrollo Tecnológico Agrario, Instituto Nacional de Innovación Agraria (INIA), Lima, Perú; b Dirección de Recursos Genéticos y Biotecnología, Instituto Nacional de Innovación Agraria (INIA), Lima, Perú; c Facultad de Ingeniería Zootecnista, Agronegocios y Biotecnología, Universidad Nacional Toribio Rodríguez de Mendoza (UNTRM), Chachapoyas, Perú

**Keywords:** Phylogenomics, Rubiaceae, chloroplast genome, quina tree

## Abstract

Here, we report the first complete chloroplast (cp) genome of *Cinchona officinalis*. This cp genome has a 156,984 bp in length with typical quadripartite structure, containing a large single copy (LSC) region (83,929 bp) and an 18,051 bp small single-copy (SSC) region, separated by two inverted repeat (IR) regions (27,502 bp). The total GC content was 37.75%. Quina tree chloroplast genome possesses 135 genes that consisted of 89 protein-coding genes, 37 tRNA, eight rRNA, and one pseudogene. Phylogenetic analysis showed that *C. officinalis* is sister to *C. pubescens* and sister to them is *Isertia laevis*; all belong to the Cinchonoideae sub-family.

Genus *Cinchona* is a member of the Rubiaceae family known for its medicinal properties as a source of quinine alkaloids that are effective against malaria (Jaramillo-Arango 1949; Andersson 1998). Among the 23 species within this genus, *C. officinalis* is known as ‘quina tree’ and represents the national tree of Peru. This species is limited to small areas in the Andean forest, and is restricted to the northern Andes in Peru (Brako and Zarucchi 1993), specifically to Cajamarca and Piura region (Huamán et al. 2019). Currently, quina tree is threatened by urban growth, farming, selective logging and massive deforestation. This tree has high capacity of regrowth in natural conditions, but only a low percentage of regeneration has been documented, suggesting low genetic diversity (Espinosa and Ríos 2017). To date, even though NGS techniques are widely used to decipher genomes, little is known about Peruvian quina tree genome characterization. In addition, knowledge about *C. officinalis* phylogenetic relationships is scarce. Therefore, in this study, we report and characterize the first complete chloroplast genome (cp) of *C. officinalis* by next-generation sequencing technology. Moreover, a phylogenetic tree of this species and its relatives is presented (Figure 1).

**Figure 1.**
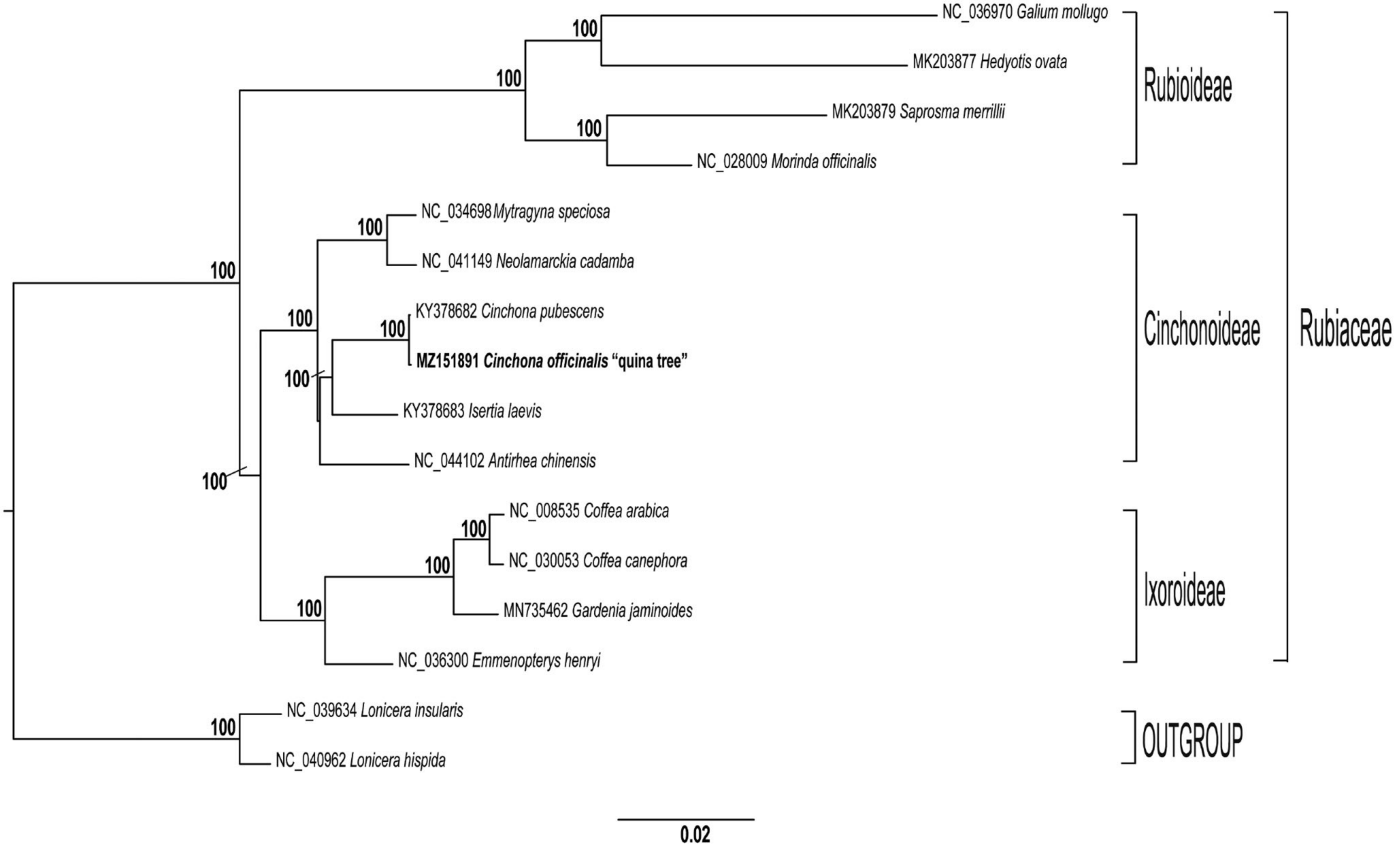
Maximum likelihood reconstruction of the 14 (including *C. officinalis*) whole chloroplast genome sequences, and two outgroups. Numbers above the branches represent bootstrap values, with only values higher than 70% shown. Names given to clades refer to the subfamilies in Rubiaceae.

We collected young fresh leaves of *C. officinalis* from Cajamarca region (6°20′58.0″S, 79°03′27.8″W) that belong to the Forestry Program of INIA. The specimen (CPUN 24126) was deposited in the Universidad Nacional de Cajamarca Herbarium. Total genomic DNA was extracted by CTAB method (Doyle and Doyle 1990) and then assessed by visualizing 900 ng on a 1% agarose gel. An Illumina pair-end (2 × 150 bp) genomic library was constructed by following the standard protocol (Illumina, USA) and sequenced using an Illumina HiSeq 2500 platform by GENEWIZ (www.genewiz.com), New Jersey, USA. Adapters and low-quality reads were removed using Trim Galore (Martin 2011). We used clean data and *Coffea arabica* (NC_008535) as a reference to assemble the chloroplast genome with the GetOrganelle v1.7.2 pipeline (Jin et al. 2020), in which SPAdes v3.11.1 (Bankevich et al. 2012), bowtie2 v2.4.2 (Langmead and Salzberg 2012) and BLAST + v2.11 (Camacho et al. 2009) were employed. Chloroplast genome was annotated with GeSeq in CHLOROBOX web service (Tillich et al. 2017).

The total length of the chloroplast genome is 156,984 bp, which is 1,795 bp longer than one of the most economically important species in the Rubiaceae family, coffee (*C. arabica*). This cp genome presents a typical quadripartite structure, containing 89,939 bp as large single copy (LSC) region and 18,051 bp as small single-copy (SSC) region, separated by two inverted repeat (IR) regions (27,502 bp), and the total GC content was 37.75%. Quina tree chloroplast genome contains 135 genes, including 89 protein-coding genes, 37 tRNA genes and 8 rRNA genes and one pseudogene. Most of these genes did not contain an intron; 18 genes harbored one intron, and two genes (pafI, clpP1) contained two introns. Most genes occurred as a single copy, except 20 genes that were duplicated in IR regions. The chloroplast genome sequence and annotation were submitted to NCBI with accession number MZ151891.

We constructed a maximum likelihood (ML) phylogenetic tree of 15 genomes obtained from GenBank. Each genome was aligned by MAFFT v7.475 (Katoh and Standley 2013). Then, we used GTR + GAMMA model of evolution to obtain the best-scoring ML tree, and then 1,000 nonparametric bootstrap inferences were performed with RAxML v8.2.11 (Stamatakis 2014). Similar to recent studies (Wikström et al. 2020), maximum likelihood analyses recovered with 100% bootstrap data three subfamilies of the Rubiaceae family. In addition, ML phylogenetic analysis showed that *C. officinalis* is sister to *C. pubescens* and sister to them is *Isertia laevis*; all belong to the Cinchonoideae subfamily (Figure 1). To our best knowledge, this is the first report of a cp genome of a plant grown in Peru. We expect this work will throw light on clarifying the evolutionary status of *C. officinalis* in genus *Cinchona.* Moreover, our next step is to continue developing molecular tools for the Peruvian national tree, promoting its adequate sustainable management, conservation and breeding.

## Data Availability

The genome sequence data that supports this study is openly available in Genbank of NCBI under the accession number MZ151891 (https://www.ncbi.nlm.nih.gov/nuccore/MZ151891). The associated Bioproject, Biosample and SRA numbers are PRJNA728344, SAMN19075496, and SRR14516337, respectively.
